# Sudden Unexpected Infant Death: Review and Analysis of Adherence to Recommendations

**DOI:** 10.7759/cureus.6076

**Published:** 2019-11-05

**Authors:** Enrique Konstat-Korzenny, Ariel Cohen-Welch, Rodrigo Fonseca-Portilla, Dan Morgenstern-Kaplan

**Affiliations:** 1 Centro De Investigación En Ciencias De La Salud Anáhuac (CICSA), Facultad De Ciencias De La Salud, Universidad Anahuac Mexico, Mexico City, MEX; 2 Centro De Investigación En Ciencias De La Salud Anáhuac (CICSA), Facultad De Ciencias De La Salud, Universidad Anáhuac Mexico, Mexico City, MEX

**Keywords:** pediatrics, sleeping practices, sids, safety, suid, safe sleeping

## Abstract

Introduction

Sudden Unexpected Infant Death (SUID) is a term that englobes the sudden and unexpected death of an infant less than 12 months, which can be explained by organic or traumatic causes, or that can't be explained such as cases of Sudden Infant Death Syndrome (SIDS). Although many risk factors have been associated with this syndrome, one of the most widely associated and studied are incorrect sleeping techniques and practices. In 2016, the American Academy of Pediatrics (AAP) updated their safe-sleeping guidelines and sleeping environment recommendations and strategies to prevent SIDS.

Methods

We conducted a cross-sectional study to analyze the sleeping environments in infants that attended both the inpatient and outpatient services at a public pediatric hospital in Mexico City. A 6-item questionnaire was applied to the parent or guardian to assess the sleeping habits of infants in their homes.

Results

A total of 184 infants were included in the study, with a mean age of 5.87 months. Overall, the number of parents that follow safe sleeping practices was very low, with no parents following all the AAP recommendations, and over 10% not following any. Although there is uncertainty about the pathogenesis of this syndrome, the focus has shifted to prevention, especially regarding the modifiable risk factors.

Conclusions

It was demonstrated that our population did not know about the proper and safe sleeping techniques. In the nearby future, our goal is for health authorities in our country to implement a strategy to make the AAP recommendations part of government health campaigns.

## Introduction

Sudden Unexpected Infant Death (SUID) is a term that englobes the sudden and unexpected death of an infant less than 12 months of age such that after a thorough investigation of the case, the cause of death was due to suffocation, entrapment, infection, ingestion, metabolic diseases, arrhythmias, trauma or Sudden Infant Death Syndrome (SIDS; term for deaths that remain unexplained even after thorough investigation of the case and scene, clinical history, and autopsy of the body) [1-2]. 

Epidemiology

According to the American Academy of Pediatrics (AAP), more than 3,500 infants die annually due to SUID and sleep-related deaths [3]. In 2010, it was reported that 1 out of 3 postnatal deaths, and 1 out of 7 infant deaths were attributed to SUID [4]. The Center for Disease Control and Prevention (CDC) reported 3,600 SUID cases in 2017, further dividing it into SIDS (38%), accidental suffocation and strangulation in bed (26%) and unknown causes (36%) [5]. The incidence of SUID seems to be more prevalent in the African American, American Indian and Native Alaskan population [3]. Since the establishment of safe-sleeping practices campaigns across the world, the incidence of SUID has dramatically fallen. An approximate decrease of 50% was reported in SUID since the mid 1980’s [6].

Pathogenesis

There is no definite pathophysiological process that can be attributed to SUID. It is thought to be a combination of various factors occuring in conjunction that lead to the lethal event. Normally, there are previous conditions that can increase the infant’s vulnerability, such as premature birth and birth defects triggering the cause of death, whether an infectious, respiratory or metabolic cause. These factors, along with an immature immunologic or nervous system, predispose the child to a higher risk of SUID [7].

Risk factors for SUID

Birth Weight and Age

Among the infant-related risk factors, low birth weight and preterm birth are the most remarkable. In one study, preterm infants had a higher risk of SUID than term infants [8]. Small for Gestational Age (SGA) infants also had a slightly increased risk of SUID (1.65 OR (95% CI 1.47 to 1.85)), while Large for Gestational Age (LGA) infants had a reduced risk of SUID (0.73 (CI 0.60 to 0.89) mortality, although the mechanisms behind these phenomena are uncertain [9].

Sleeping Position

Sleeping position is another important risk factor and the main focus of many SUID prevention campaigns [6,10]. Non-supine or prone sleeping positions are associated with a higher risk of SUID [3,11]. One study that analyzed 11,717 infant death cases, where 27.4% classified as SUID, prone sleeping was reported in up to 42.4% of the deaths [12]. A recent study gathered polysomnographic information, documenting that infants during prone sleeping had a higher heart rate, decreased oxygen saturation and more time with oxygen saturation below 90%, compared with infants who were sleeping in the supine position, offering possible explanations behind the physiopathology of inadequate sleeping positions and their relation to SUID [13].

Sleeping Environment

Loose bedding or objects in the immediate sleeping environment of the infant are also associated with SUID. One study reported that 14% of SUID cases reported by the CDC from 2011-2014 were attributed to suffocation, and 69% of those deaths were attributed to loose bedding [14]. One systematic review gathered information about 10 SUID studies and concluded that almost 25% of cases were related to loose bedding [15].

Sharing beds with another person (bed sharing) and co-sleeping are common practices throughout the world, especially in underdeveloped countries. While some benefits have been reported, mainly related to improved breastfeeding and increased emotional bond between mother and infant [16-18], many experts and studies advise against it because of its association with SUID. A meta-analysis concluded that co-sleeping increased the risk of SUID, even if parents did not smoke. [19] Various other studies and meta-analysis have demonstrated an increased risk with these practices [12,20]. Sharing a sleeping surface, other than a bed, such as a sofa, has also been studied and presents a high risk of SUID [21].

Maternal or Environmental Risk Factors

Smoking during pregnancy or after birth is another important risk factor for SUID [20]. A twofold increase in SUID risk was reported in infants born to mothers who smoked during pregnancy, as well as a linear increase in the risk of SUID with each additional cigarette smoked during pregnancy [22]. An increased risk was also reported in infants whose parents were passive smokers [23]. In fact, any drug exposure during pregnancy, including alcohol, is associated with an increased risk for SUID [3]. Other risk factors include young maternal age (< 20 years old).

Protective Factors for SUID

Breastfeeding

Breastfeeding has been shown to reduce the risk of various diseases including otitis media, necrotizing enterocolitis, asthma, diabetes, obesity, and SUID [24]. One meta-analysis demonstrated that breastfeeding for at least two months was protective for SUID, with a directly proportional relation with increased time of breastfeeding [25]. Partial and exclusive breastfeeding proved to be protective in infants who only slept in the supine position, although exclusive breastfeeding proved more protective than partial [20]. Because of all these described benefits, the term “breastsleeping” was coined. It tries to englobe the notion that maternal contact has a critical role in optimization of breastfeeding, and the fact that data and information should be gathered from studies of breastsleeping dyads, because of possible different outcomes from studies where no breast sleeping is performed [26].

Room Sharing

Room sharing is not a synonym of co-sleeping. It implies sleeping in the same room but on separate surfaces, albeit close to them. Studies have demonstrated that room sharing may reduce the risk of SUID in half. Therefore, room sharing is essential for at least the first six months of life and recommended ideally during the first year [27].

Pacifier Use

The use of a pacifier before naptime and bedtime is accepted and recommended by the AAP, even though it may be related to cases of malocclusion and breastfeeding technique concerns. Multiple studies have yielded evidence that this intervention may reduce the risk of SUID from 50-90% [27-28].

Current recommendations

In a 2016 update, the American Academy of Pediatrics published their new recommendations regarding safe-sleeping techniques to reduce the risk of SUID, emphasizing the need for supine positioning during sleep. The summary of these recommendations are outlined in the questionnaire below [3].

## Materials and methods

A cross-sectional study was performed in a second level, public, pediatric hospital in Mexico City, from May the 1st to July 30th of 2019. Subjects were selected via convenience sampling and data was collected by interviewing infant’s parents or legal guardians. A series of specific questions were asked based on the AAP 2016 guidelines (Table 1), to depict whether the studied population followed the established recommendations or not. We focused on the most significant infant-related risk factors, since most of the maternal-related risk factors can't be controlled after the birth of the infant. Parents gave consent and agreed to participate in the direct verbal questionnaire.

**Table 1:** AAP 2016 guidelines with strength of recommendation to prevent SUID [3] AAP: American Association of Pediatrics. SUID: Sudden Unexpected Infant Death CDC: Centers for Disease Control NICU: Neonatal Intensive Care Unit

American Academy of Pediatrics Safe-Sleeping Recommendations	
A-level recommendations	Back to sleep for every sleep
	Use a firm sleep surface
	Breastfeeding is recommended
	Room sharing with the infant on separate sleep surfaces
	Keep soft objects and loose bedding away from the sleep area
	Consider offering a pacifier at bedtime and naptime
	Avoid smoke exposure during pregnancy and after birth
	Avoid alcohol and illicit drug use during pregnancy and after birth
	Avoid overheating
	Pregnant woman should seed and obtain regular prenatal care
	Infants should be immunized in accordance with AAP and CDC recommendations
	Do not use home cardiorespiratory monitors as a strategy to reduce the risk of SIDS
	Health care providers, staff in newborn nurseries and NICU’s, and childcare providers should endorse and model the SIDS risk-reduction recommendations from birth
	Media and manufacturers should follow safe sleep guidelines in their messaging and advertising
	Continue the “Safe to Sleep” campaign, focusing on ways to reduce the risk of all sleep-related infant deaths, including SIDS, suffocation and other unintentional deaths. Pediatricians and other primary care providers should actively participate in this campaign
B-level recommendations	Avoid the use of commercial devices that are inconsistent with safe sleep recommendations.
	Supervised, awake tummy time is recommended to facilitate development and minimize development of positional plagiocephaly.
C-level recommendations	Continue research and surveillance on the risk factors, causes and pathophysiologic mechanisms of SIDS and other sleep-related infant deaths, with the goal of eliminating these deaths entirely.
	There is no evidence to recommend swaddling as a strategy to reduce the risk of SUID

Inclusion Criteria

To be included in the study, subjects had to be at least 1 day old, and less than 12 months old. We included all patients that presented in the out-patient clinic, in-patient hospitalization service, as well as those who presented to the emergency room.

Exclusion Criteria

Subjects older than 12 months were excluded from the study and those whose parents or legal guardians refused to answer the questionnaire. Patients with special care or nighttime monitoring were excluded as well.

Data was collected with a questionnaire encompassing infant-related risk factors for SUID, and it was answered by the parent or legal guardian of the infant. We asked the patient’s legal guardian questions based on the known risk factors for SUID (Table 2), such as co-sleeping, sleeping in a supine position, the use of pillows, sleeping environment characteristics, bedding and the use of a pacifier.

**Table 2:** Summary of SUID maternal and infant-related risk factors SUID: Sudden Unexpected Infant Death

SIDS Risk Factors	
Maternal	Smoking during or after pregnancy
	Maternal age <20 years
	Inconsistent prenatal care
Infant	Prone or side sleep position
	Loose bedding
	Bed-sharing
	Low birth weight and prematurity
	Siblings with SUID

Statistical Analysis

The data collection was made in Microsoft Excel 2018 and further statistical analysis was performed in SPSS version 21.0 (IBM), and data for categorical variables is reported in frequencies and percentages, the numerical variables are expressed in means and standard deviations.

## Results

A total of 184 infants were included in the analysis. The mean age of the study sample was 5.87 months (SD 3.18). There were 117 males, and 67 females. Of the 184 infants surveyed, the vast majority did not sleep alone, contrary to the AAP recommendations for safe sleeping (78.8% did not sleep alone). Additionally, more than half of the infants did not sleep in a supine position, putting them at risk for SUID (51.1% did not sleep supine) (Table 3).

**Table 3:** . Results from the questionnaire looking for known risk factors for SUID and incidence of falls from bed. SUID: Sudden Unexpected Infant Death

Questionnaire Answers		
	Frequency (n=184)	Percentage
Sleep Alone	39	21.20%
Use Pillow	117	64%
Stuffed Animals	18	9.80%
Sleep Supine	90	48.90%
Falls	24	13%
Use Pacifier	5	2.70%

**Figure 1:**
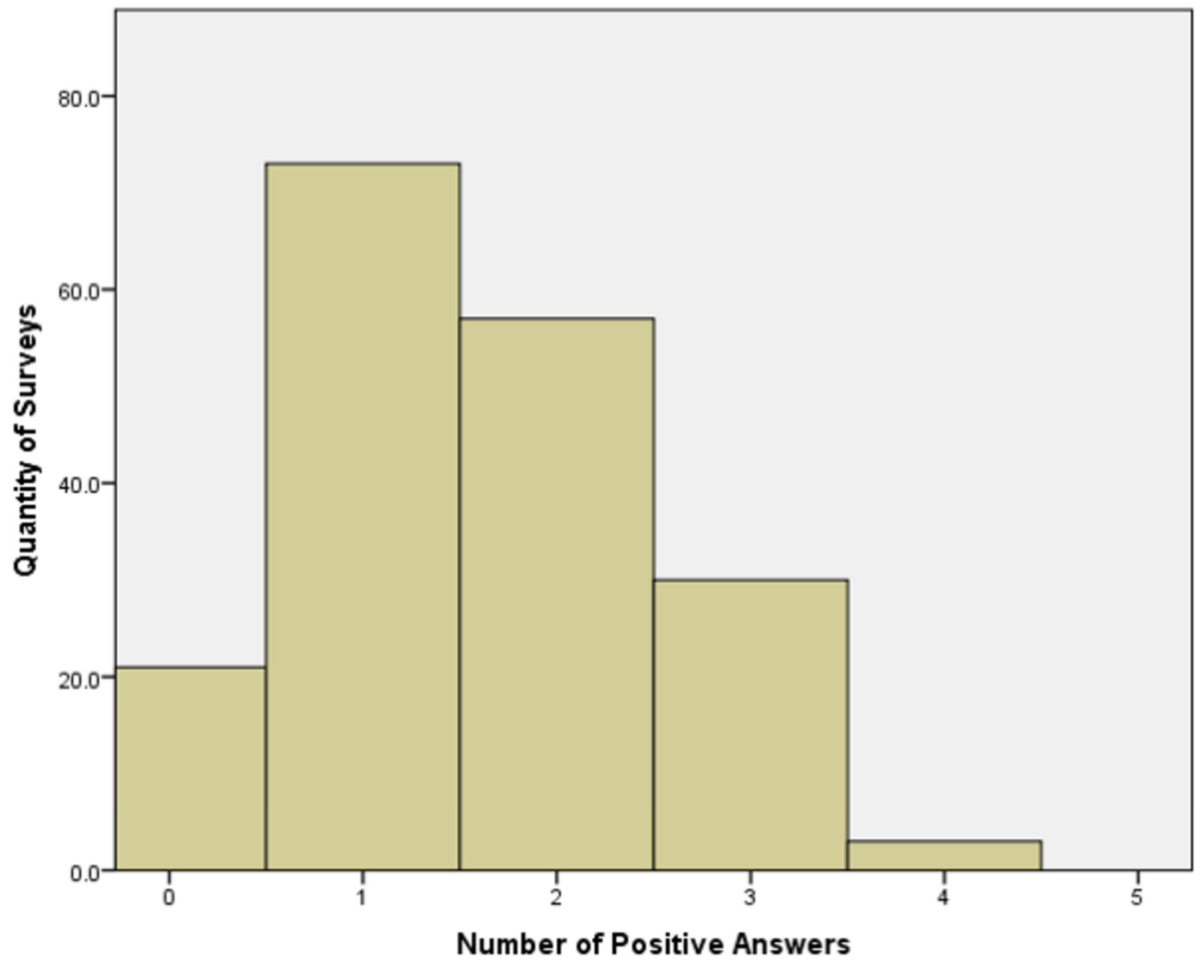
Histogram showing the frequencies of each number of positive answers (amount of AAP recommendations followed) detected by the 6-question survey done in the present study. AAP: American Association of Pediatrics

Overall, the amount of patients that follow safe sleeping practices in our sample was low, the mean number of positive answers in the questionnaire was 1.57 (SD 0.9), with no patients following all the recommendations in the questionnaire, and over 10% following no recommendations at all (Figure 1).

## Discussion

SUID remains an important public health problem, due to the high death burden. Many cases could be prevented if the corresponding risk factors had been avoided. Therefore, several strategies have been addressed, mainly by developed countries, to reduce the number of affected children. The data we found in our study is alarming, and the problem must be addressed by national health authorities.

Although there is uncertainty about the pathogenesis of this syndrome, the focus has shifted to prevention, especially of the modifiable risk factors, through specific campaigns, recommendations and guidelines by pediatric associations, based on extensive observation and reported studies. Regarding our work, the results obtained were not far from what we hypothesized in a low socioeconomic status population, which was the case of the sample obtained in this particular hospital. Other studies with similar populations had similar results regarding bed sharing and co-sleeping prevalence [29]. Like what other studies have found, our population could extrapolate to the findings in the African American, American Indian and Native Alaskan population, where similar sleeping customs are practiced [3]. However, there is no clear epidemiological data on this pathology in our country. There seems to be a correlation with better sleep practices in mothers with higher socioeconomic and educational background [30] which could be assessed in further studies with a different population. 

It is important to note that after every interview with the parent or legal guardian, we explained the current recommendations and guidelines to the parents, the actions to avoid and the actions to implement. However, many of these current guidelines (such as avoiding co-sleeping with the infant, which is very prevalent in the Mexican population) are unpractical for our population, mainly due to social, economic and psychological situations, and changing this paradigm will be a challenge. Furthermore, safe sleeping campaigns must be conducted in our country to instruct this knowledge to current and future parents, preventing cases of SUID. Medical personnel will have to shift their focus to inform and make parents aware of these recommendations. Recommendations are relatively simple for most parents to follow, but the lack of information and knowledge on this topic, both by parents and health workers make it hard to understand their importance. 

Up to this date, there is not a consensus or guideline regarding risk reduction strategies for SUID in our home country, and therefore, the population has never been informed of the possible interventions to reduce the risk, and believe that their current sleeping practices are safe and adequate.

## Conclusions

It was demonstrated that our population did not know about the proper and safe sleeping techniques to avoid the known sleep-related risk factors associated with SUID. Unfortunately, this is still an understudied subject in our country and in the world. Mexico lacks concluding and reliable epidemiological information to measure the real prevalence and incidence of this public health problem. With this study we were able to demonstrate the impending need to implement a “Safe-Sleeping” campaign in our country, such as the ones carried out in other parts of the world, where there is conclusive evidence of the benefits of implementing such campaigns to prevent SUID in the future. Optimistically in a nearby future, health authorities in Mexico and other Latin-American countries will implement a strategy to make the AAP recommendations part of government health campaigns parallel to those of universal vaccination of children and exclusive breastfeeding for the first six months of life.
